# Relationship between reward-related evoked potentials and real-world motivation in older people living with human immunodeficiency virus

**DOI:** 10.3389/fnagi.2022.927209

**Published:** 2022-09-01

**Authors:** Gloria Castaneda, Ana-Lucia Fernandez Cruz, Marie-Josée Brouillette, Nancy E. Mayo, Lesley K. Fellows

**Affiliations:** ^1^Department of Neurology and Neurosurgery, Faculty of Medicine and Health Sciences, Montreal Neurological Institute, McGill University, Montreal, QC, Canada; ^2^Department of Psychiatry, Faculty of Medicine and Health Sciences, McGill University, Montreal, QC, Canada; ^3^Division of Clinical Epidemiology, Department of Medicine, Faculty of Medicine and Health Sciences, McGill University, Montreal, QC, Canada

**Keywords:** HIV/AIDS, electroencephalography, apathy, biomarkers, feedback

## Abstract

Apathy, a clinical disorder characterized by low motivation, is prevalent in people living with Human Immunodeficiency Virus (HIV). It affects mental and physical health-related quality-of-life, medication adherence, and is associated with cognitive decline. However, the causes of apathy and the underlying brain mechanisms in HIV are unknown. Brain responses to reward may be relevant to understanding apathy and might serve as biomarkers for diagnosis or treatment response. Electroencephalogram (EEG) responses to gain and loss feedback in simple guessing tasks have been related to apathy in neurodegenerative conditions and healthy individuals. The primary aim of this study is to contribute evidence regarding the relationship between two EEG correlates of reward processing, the Reward Positivity, and the Feedback-P300, and real-world motivated behavior indicated by self-reported hours engaged in goal-directed leisure activities per week, in older individuals with well-controlled HIV infection. High-density EEG was collected from 75 participants while they performed a guessing task with gain or loss feedback. We found that a later component of reward processing, the Feedback-P300, was related to real-world engagement, while the earlier Reward Positivity was not. The Feedback-P300 measured with EEG holds promise as a biomarker for motivated behavior in older people living with HIV. These findings lay the groundwork for a better understanding of the neurobiology of apathy in this condition.

## Introduction

Apathy, a multifaceted syndrome characterized by reduced goal-directed behavior, is frequent in older people living with HIV, even with combination antiretroviral therapy (cART) (McIntosh et al., 2015) and is associated with poor health-related quality of life (Barclay et al., 2007; Kamat et al., 2012, 2016) and worse cART adherence (Babicz et al., 2020). However, little is known about the biological basis of apathy symptoms in HIV. Recent work has shown that self-reported apathy is not associated with plasma biomarkers of inflammation (Woods et al., 2022), arguing for neuropsychological rather than systemic causes.

Neuroscience research has proposed conceptual frameworks that dissect apathy into specific components, with reward-related processes of particular interest (Fellows, 2004; Husain and Roiser, 2018; Barch et al., 2019). Anticipation and receipt of reward following a motor response triggers a series of neural and neurochemical events including dopamine release and changes in frontostriatal signaling believed to be important in energizing goal-directed behavior and adjusting those behaviors in response to feedback. Electroencephalography (EEG) can be used to assess these processes in the human brain (Glazer et al., 2018). The feedback-P300 (FB-P3) and the Reward Positivity (RewP) are the most widely studied feedback-related event-related potentials (ERP). These occur at different time points; both differ in amplitude following gains (reward) compared to losses (punishment) (Sato et al., 2005; San Martín, 2012; Glazer et al., 2018; Krigolson, 2018). There is some evidence in other populations that one or both of these potentials may index clinically relevant neural activity, with individual differences in ERP amplitudes relating to apathy, as well as to related constructs of depression and anhedonia. The FB-P3, but not the RewP, was related to self-reported apathy in healthy young adults (Takayoshi et al., 2018), while the RewP was associated with apathy in Parkinson’s Disease (PD) (Martinez-Horta et al., 2014). However, both of these studies involved very small samples. Despite the prevalence and quality of life impacts of apathy in HIV, and the evidence that frontal lobe and striatal dysfunction are common in people living with chronic, treated HIV (Israel et al., 2019), EEG has yet to be applied to study motivated behavior in this population.

Here, we contribute evidence regarding the relationship between the amplitudes of two EEG potentials elicited by feedback, the RewP and FB-P3, and two indicators of motivation: self-reported time engaged in goal-directed leisure activities, and self-reported apathy indicated by selected items from a widely used apathy questionnaire (Starkstein Apathy Scale), in older people with HIV, without current substance use disorder. Participants were drawn from an on-going longitudinal study of brain health in HIV in Canada. We hypothesized that the conditional waveform for gain feedback for both ERPs will be positively associated with hours spent on meaningful activity. All hypotheses and analyses were pre-registered.^1^

## Materials and methods

### Participants

Eighty-five people with well-controlled HIV consented to participate in this study. Ten participants could not contribute EEG data to the analysis due to technical problems in EEG acquisition (*N* = 3) or EEG signal of insufficient quality to allow the ERPs of interest to be reliably estimated, leaving a sample of 75 participants. Data were collected as part of a baseline assessment for two pilot randomized trials of interventions to improve cognition (physical exercise or computerized cognitive training). These were sub-studies sampling from the Positive Brain Health Now (BHN) cohort, a longitudinal study of brain health in older individuals living with HIV recruited from specialized HIV clinics at 5 sites in large urban centers across Canada (Mayo et al., 2016).

Inclusion criteria in the main BHN cohort were age 35 years or older, HIV infection for at least 1 year, and ability to communicate in French or English. Exclusion criteria included clinically diagnosed dementia severe enough to preclude informed consent, life expectancy less than 3 years, non-HIV-related neurological disorders likely to affect cognition, psychotic disorder, current substance use disorder or severe substance use disorder within 12 months prior to cohort enrollment, active CNS opportunistic infection, or hepatitis C on interferon treatment. Participants in the cognitive training trial also required access to the Internet, while those in the physical exercise trial reported sedentary behavior (i.e., moderate physical activity for no more than 30 min and no more than twice a week) and were excluded if they had cardiovascular or musculoskeletal contraindications for vigorous exercise. The protocol was approved by the Research Ethics Board of the McGill University Health Center and all study participants provided written informed consent.

### Real-world motivated behavior

Participants reported the number of hours they spent in a typical week on goal-directed leisure activities, including reading, checking their email, surfing the internet, crafts or hobbies, other computer-based activities such as games, or other leisure activities they specified. This list was derived from the Community Healthy Activities Model Program for Seniors (CHAMPS) (Stewart et al., 2001). Responses were summarized as the total number of hours spent on these activities per week. Based on a prior study relating apathy to a neurobehavioral test of motivated behavior in a different HIV + sample (Castaneda et al., 2021), this was pre-registered as the primary outcome measure. To allow the present work to be related to the wider literature, participants also completed the Starkstein Apathy Scale- Rasch version (SAS-R) (Starkstein et al., 1992; Starkstein and Leentjens, 2008). Eight items were administered from the standard SAS and re-scored based on a Rasch analysis performed on this data (following the approach described in Hum et al., 2021). Scores ranged from 0 to 100, where higher values indicate higher motivation.

### Electrophysiological measures

**FIGURE 1:**
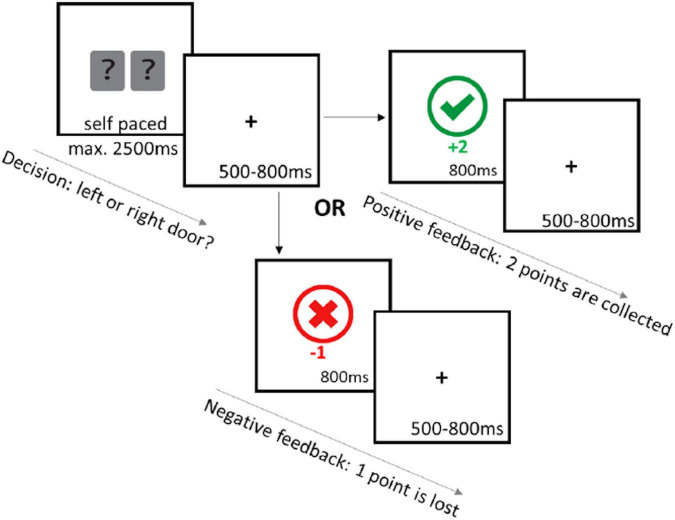
Schematic showing the guessing task. Participants were presented with two doors for 2,500 ms where they are asked to select a door with the “RIGHT ARROW” to select the right door or the “LEFT ARROW” to select the left door. A fixation cross with a jittered 500–800 ms interval was presented after selection of the door. If the “correct” door was selected participants earned two points and a green check mark appeared. If the “incorrect” door was selected, they lost one point and a red cross appeared. Next trial continues after a jittered 500–800 ms interval.

The guessing task used here was a modified version of the Doors task (Gehring, 2002; Martinez-Horta et al., 2014), with 150 trials divided into 5 blocks. A trial began with the presentation of the stimuli for 2,500 ms. The participant selected a door with the “right arrow” or the “left arrow” key, using their index fingers. A fixation cross was then presented in the center of the screen followed by feedback presentation for 800 ms. Gain feedback was worth two points, signaled by a green checkmark, whereas loss feedback was the loss of one point, signaled by a red “x.” The next trial began with the presentation of a fixation cross with a duration randomly selected from the following times: 550, 650, 750, 850, 950, or 1,590 ms. All trials had a 50% probability of gain or loss feedback, regardless of the choice made. This was unknown to participants, who were instructed to “try your luck to choose the winning door” and were encouraged to collect as many points as possible. Point totals were presented every 30 trials to keep participants engaged (Figure 1).

### Electroencephalographic data acquisition and analysis

A 256-channel high impedance HydroCel Geodesic Sensor Net (Electrical Geodesics, Inc., Eugene, OR) with NetStation 5 software was used to record the EEG data. Electrode impedance was kept below 50 kΩ using Cz as a reference. Online visualization filters were 60 Hz for Notch, 5 Hz for high-pass, and 120 Hz for low-pass. The 78 electrodes located on the neck and cheeks were contaminated with muscle artifact and were removed in all participants before data pre-processing.

EEG data were processed offline for all subjects using Brainstorm (Tadel et al., 2011). EEG recordings were filtered using a bandpass filter of 0.1–30 Hz, down-sampled to 500 Hz, and re-referenced to the right and left mastoid electrodes. Bad channels were identified with power spectrum density plots using Welch’s method and raw signal manual examination. Segments higher than 200 μV were discarded. Automatic blink detection was conducted on the four electrodes located above and below each eye and artifact correction was performed with Signal-Space Projection. A baseline correction of -200 ms before feedback onset was applied. Epochs with an activity of ± 100 μV were rejected. Trials with missing responses or responses with a reaction time of less than 5 ms were removed. The average number of trials included for gain and loss feedback was *N* = 74 (*SD* = 6.1) and *N* = 74 (*SD* = 6.2), respectively.

## Analysis

### Event-related potentials analysis

The RewP (200–300 ms) was measured at two clusters of electrodes centered at FCz (i.e., E015, E006, E023; Luu and Ferree, 2005), where it typically peaks (Miltner et al., 1997; Holroyd and Krigolson, 2007), and Fz (E021, E013, E028), a site used in previous studies (Hajcak et al., 2005). The FB-P3 (300–600 ms) was measured at two cluster of electrodes centered at Cz (E081, E045, E132) and Pz (E101, E129, E100), where it typically peaks (Hajcak et al., 2005; Polich, 2007; Kleih et al., 2010; Peterburs et al., 2013). The N1 (90–120 ms), an ERP reflecting early sensory processing (Luck et al., 2000) here served as a check on the specificity of the hypothesized relationships, and was measured at a cluster centered at Cz (E081, E045, E132), where it is typically maximal (Debruille et al., 2019).

### Statistical analyses

The main effects of feedback condition on the RewP and FB-P3 were tested using a repeated-measures ANOVA with two factors: condition (gain, loss) and clusters. Multiple linear regression was conducted to assess the contribution of the gain conditional waveform on predicting the primary real-world motivation measure, i.e., hours of meaningful activities. Given that age influences EEG signal, age was included in the models (Rossini et al., 2007; Gajewski et al., 2018). The same regression model was applied to explore the relationship between ERP amplitudies and the secondary motivation measure, SAS-R.

Additional exploratory analyses were conducted to test whether the effects observed at clusters were also present at single electrodes that are more typically reported in the literature, which has mainly used 64-channel EEG (FCz = E015, Fz = E021, Pz = E101, Cz = E081). We also explored the contribution of the ΔRewP, and the loss conditional waveform on predicting each of the two outcome measures of real-world motivation. To explore the contribution of HIV infection severity to the observed variation in ERP amplitudes, we conducted a regression analysis testing the effect of nadir CD4 as an indicator of HIV infection severity on RewP and FB-P3, with age included in the models.

## Results

### Participant characteristics

Demographic and clinical characteristics of the sample are presented in Table 1. Ninety-two percent of participants were taking cART at the time of the study.

**TABLE 1:** Demographic and clinical characteristics of the sample (*N* = 75).

Characteristics	M or %	SD	Median
Age (years)	54.84	6.99	53.80
Men	89%		
Women	11%		
Education (years)	12.97	3.30	12.00
Duration of HIV infection (years)	17.81	7.22	18.00
Current CD4 cell count (cells/μL)	649.17	256.04	640.00
IQR	481-836		
0–199	4.33%		
200–500	21.33%		
>500	73.33%		
Nadir CD4 cell count (cells/μL)	213.62	157.33	170.50
IQR	133–256		
**Plasma viral load**			
Virologically suppressed (≤ 50 copies/mL).	93.33%		
**Self-reported measures**			
Meaningful activity [h/week]	33.22	23.07	31.00
SAS-R (Motivation) [0–100]	52.04	13.83	49.98
HADS-D (Depression) [0–21]*^a^*	15.84	4.01	17.00
HADS-A (Anxiety) [0–21]*^a^*	13.99	4.38	14.00
**Performance measure**			
B-CAM (Cognitive performance) [0–35]	20.42	4.23	20.50

In all self-reported measures higher scores indicate more motivation. IQR, Interquartile range; SAS-R, Starkstein Apathy Scale-Rasch; HADS, Hospital Anxiety and Depressive Scale; B-CAM, Brief Cognitive Ability Measure.

^a^Scores were reversed so that higher scores indicate fewer symptoms.

### Feedback-related evoked potentials

**FIGURE 2:**
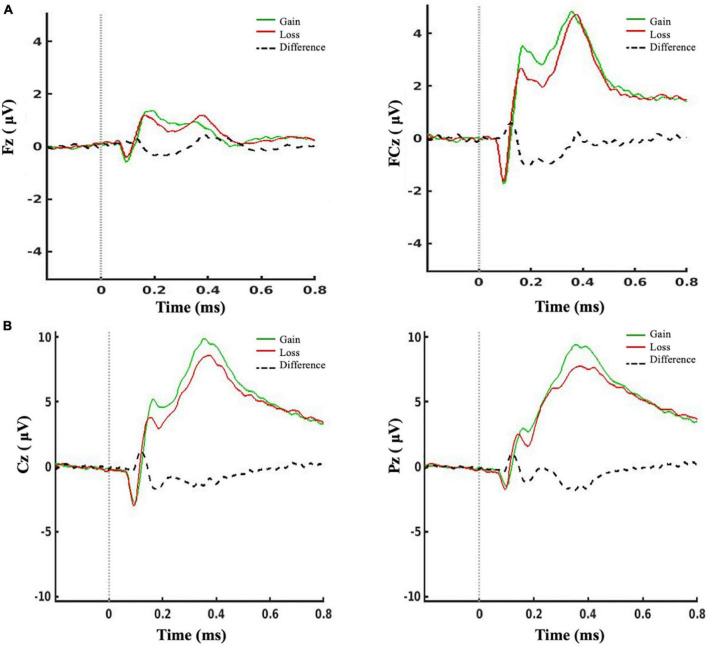
ERPs for gain feedback (in green), loss feedback (in red) conditions, and the mean difference of loss minus gain feedback (black dotted line). **(A)** The RewP (200–300 ms after feedback presentation) was measured at a frontal and a frontocentral cluster. **(B)** The FB-P3 (300–600 ms after feedback) was measured at a central and a centroparietal cluster.

Participants completed an average of 99.8% (*SD* 0.01) of trials. There was a significant effect of feedback condition [*F*(1, 74) = 65.14, *p* < 0.001, η^2^ = 0.47] and cluster site [*F*(1, 74) = 25.87, *p* < 0.001, η^2^ = 0.26] and a significant interaction [*F*(1, 74) = 26.96, *p* < 0.001, η^2^ = 0.27] on the mean amplitude of the RewP. *Post hoc* comparisons (Bonferroni-corrected) revealed that the signal at the FCz cluster site was significantly larger than at Fz (*p* < 0.001), as seen in Figure 2, and the mean amplitude of the RewP was significantly larger for gain than for loss feedback (*p* < 0.001).

There was a significant effect of feedback condition [*F*(1, 74) = 7.18, *p* = 0.009, η^2^ = 0.09] on the mean amplitude of the FB-P3. There was no effect of cluster site, nor interaction. *Post hoc* pairwise comparisons (Bonferroni-corrected) revealed that the mean amplitude of the FB-P3 was significantly greater for gain than for loss feedback (*p* = 0.029). There was also a significant effect of feedback type on N1 amplitude [*F*(1, 73) = 13.68, *p* < 0.001, η^2^ = 0.16], with the amplitude significantly larger for gain than for loss feedback (*p* < 0.001) (Figure 2). Note that the N1 could not be reliably estimated in one participant.

### Relationships between gain feedback and real-world motivation

**FIGURE 3:**
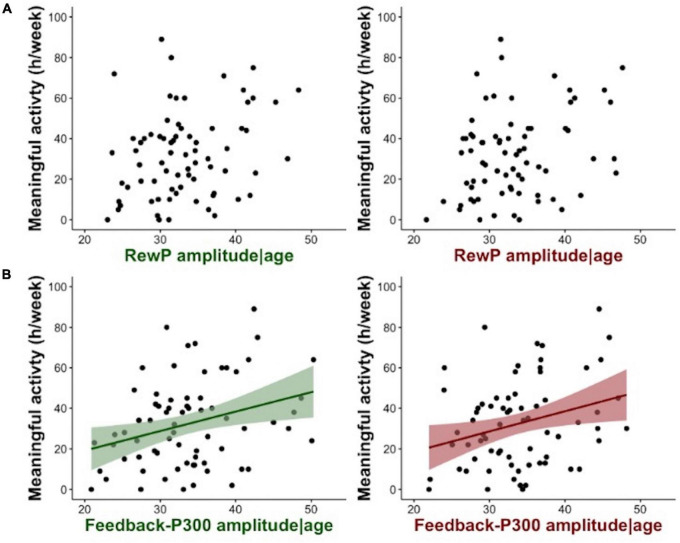
**(A)** Shows scatterplots of the relation between RewP gain feedback condition (in green) or RewP loss feedback condition (in red) adjusted for the effects of age, and time spent on meaningful activity in a week. **(B)** Shows the relation between FB-P3 gain feedback condition (in green) or FB-P3 loss feedback condition (in red) adjusted for the effects of age, and meaningful activity. Shading shows the 95% confidence intervals.

There was a significant relationship between hours of meaningful activity and the amplitude of the FB-P3 at: Cz [*F*(2, 71) = 3.74, *p* = 0.029, adj *R*^2^ = 0.070] and Pz [*F*(2, 71) = 4.23, *p* = 0.018, adj *R*^2^ = 0.082], with no effect of age at either cluster site (*p* > 0.1) (Table 2). In contrast, there was no significant relationship between the RewP gain conditional average at Fz [*F*(2, 71) = 0.70, *p* = 0.501, adj *R*^2^ = -0.008] or FCz [*F*(2, 71) = 2.35, *p* = 0.103, adj *R*^2^ = 0.036] and hours of meaningful activity (Figure 3). Meaningful activity was also not predicted by the N1 amplitude [*F*(2, 70) = 0.75, *p* = 0.477, adj *R*^2^ = −0.007]. Exploratory analysis of single electrodes confirmed the results from the cluster analysis, i.e., only the amplitude of the FB-P3 at Pz was significantly related to meaningful activity (*p* = 0.009), with no effects of age (*p* > 0.6); [*F*(2, 71) = 4.11, *p* = 0.020, adj *R*^2^ = 0.079]. No significant relationships were found between the RewP (*p* > 0.2), FB-P3 (*p* > 0.4), or N1 (*p* > 0.6) gain conditional averages and self-reported motivation (SAS-R) (Supplementary Table 1).

**TABLE 2:** Multiple linear regressions predicting real-world motivation (meaningful activities).

Outcome: Meaningful activities, 33.22 (23.07)	Parameter estimate (β)	Standard error	95% CI (lower bound, upper bound)	*R* ^2^
Predictors				
**Gain feedback**				
RewP amplitude, μV	2.15	1.12	(−0.08, 4.38)	
Age, decades	–2.83	3.82	(−10.44, 4.78)	0.06
FB-P3 amplitude, μV	2.06**	0.76	(0.55, 3.57)	
Age, decades	–1.64	3.78	(−9.17, 5.89)	0.11*
N1 amplitude, μV	–0.86	1.20	(−3.25, 1.53)	
Age, decades	–4.47	3.98	(−12.41, 3.47)	0.02
**Loss feedback**				
RewP amplitude, μV	2.16	1.11	(−0.05, 4.38)	
Age, decades	–3.43	3.79	(−10.98, 4.13)	0.06
FB-P3 amplitude, μV	1.92**	0.77	(0.39, 3.46)	
Age, decades	–2.30	3.77	(−9.82, 5.22)	0.09*
N1 amplitude, μV	0.01	0.94	(−1.87, 1.89)	
Age, decades	–3.84	4.15	(−12.11, 4.32)	0.01
**Difference (loss-gain)**				
ΔRewP, μV	0.17	2.60	(−5.03, 5.36)	
Age, decades	–3.82	3.95	(−11.69, 4.06)	0.01

Results are from a cluster centered at FCz for RewP and a cluster centered at Pz for FB-P3 gain and loss feedback conditional averages. The ΔRewP is calculated as loss minus gain feedback. To facilitate interpretation, age is expressed in decades.

*p < 0.05; **p < 0.01.

### Relationships between loss and feedback difference and real-world motivation

Time spent on meaningful activity was also predicted by the amplitude of the FB-P3 loss conditional average at clusters Cz [*F*(2, 71) = 3.76, *p* = 0.028, adj *R*^2^ = 0.070] and Pz [*F*(2, 71) = 3.62, *p* = 0.032, adj *R*^2^ = 0.067], with no effect of age (Table 2). Meaningful activity was not significantly predicted by the RewP loss conditional average or the ΔRewP at either cluster site. None of the ERPs were related to self-reported apathy (SAS-R) (Supplementary Table 1).

### Relationship between feedback-evoked potentials and other characteristics of the sample

Neither gain or loss feedback conditional waveforms, nor their difference were predicted by nadir CD4 cell count (Supplementary Table 2). Meaningful activity and SAS-R were not significantly correlated (*r* = 0.14, *p* = 0.25). Given that other brain health constructs such as depression, anxiety or cognitive impairment might influence motivated behavior, we tested for such relationships. Global cognitive ability was assessed with a brief computerized cognitive test battery (Brouillette et al., 2015), and self-reported anxiety and depression were assessed with the Mental Health Index (Zigmond and Snaith, 1983) in the parent study. Only cognitive ability was significantly related to hours of meaningful activity (*r* = 0.28, *p* = 0.02).

## Discussion

This study found that individual differences in the amplitude of the FB-P3, an EEG potential evoked by feedback that has been studied extensively in other conditions, was related to time engaged in real-world motivated behavior in older people living with HIV in Canada. This ERP also has been linked to individual differences in self-reported apathy in healthy young people (Takayoshi et al., 2018). While P3 responses can be elicited by a variety of events, and may reflect several cognitive processes, the P3b elicited by motivational feedback, and with a temporoparietal topography, has been proposed to relate to learning and memory processes heavily engaged in tasks providing trial-by-trial feedback (Polich, 2007; Balconi and Crivelli, 2010; Palidis et al., 2019). Although EEG is not suited to establishing the underlying brain structures, its excellent temporal resolution provides insights into the processes that may be disrupted in people with HIV who are less engaged in real-world motivated behavior. Our findings suggest temporal specificity of the observed relationship: neither the very early N1 response to the visual stimulus signaling feedback, nor the later RewP waveform related to real world behavior. This is in line with the one study of apathy on healthy participants (Takayoshi et al., 2018) but in contrast with a study in Parkinson’s Disease (Martinez-Horta et al., 2014). These discrepancies may be due to small samples, differences in the details of the task, or differences in the signal-to-noise of the ERPs, but it is also likely that apathy has different neural mechanisms in different clinical populations. Further work on more fully defining the behavioral and neural correlates of apathy at the component process level will be important in developing more specific, neuroscience-informed models of disturbances in goal-directed behavior across clinical conditions.

Neurobehavioral difficulties in people living with chronic HIV may be due directly to “legacy” viral effects on the brain, indirectly due to comorbidities such as cerebrovascular injury, psychosocial factors (Brew et al., 2009; Sanford et al., 2018b; Cysique and Brew, 2019; Lam et al., 2019; Fernandez Cruz et al., 2021), or a complex interplay betwen several of these variables (Mayo et al., 2020). In a recent study from our group that focused on cognition, in an overlapping sample, a portion of the variance in the oddball-evoked P300 amplitude was explained by nadir CD4 cell count, an indicator of HIV infection severity (Fernandez Cruz et al., 2021). Here, variation of the FB-P3 was not explained by nadir CD4 count. These contrasting findings suggest that the P300 elicited by oddball tasks and the FB-P3 in this feedback task likely reflect activity in distinct neural circuits, differently susceptible to direct HIV-related injury. This is supported by the wider literature. The oddball-evoked P300 is thought to be a P3a response, linked to prefrontal, frontal, and anterior temporal regions, while the brain regions that have been suggested to generate the P3b (i.e., generated by feedback), are posterior temporal, parietal, and posterior cingulate (Conroy and Polich, 2007; Spyrou and Sanei, 2008). Direct effects of HIV at the time of initial or untreated infection may preferentially affect the fronto-striatal systems thought to underpin the oddball-evoked P3a (Plessis et al., 2014). The brain basis for the variation in the FB-P3 linked to real-world engagement remains to be established. Candidates include co-morbidities common in HIV that also affect the brain, such as cerebrovascular injury. This would be a fruitful direction for future work, as it might suggest specific lifestyle or other interventions relevant to improving real-world motivated behavior in HIV.

Although a reduction in goal-directed behavior is a *sine qua non* of apathy (Starkstein et al., 1992; Starkstein and Leentjens, 2008), we did not find a relationship between EEG responses and apathy reported on a set of items from the Starkstein Apathy Scale in the current study, selected from the full scale based on the strength of their measurement properties. Even with this refinement, recent work from our group has suggested that real-world engagement may be a more suitable indicator of apathy or motivation than the SAS, at least if the goal is to identify neural or neurobehavioral correlates (Castaneda et al., 2021). The lack of a relationship between real-world activities and SAS-R score in the current sample raises further question about the ecological validity of the SAS in HIV. We have also identified psychometric limitations of the SAS in stroke patients (Hum et al., 2021).

The number of weekly hours spent on activities that are personally meaningful is a measure based on the CHAMPS, often used in clinical assessments in occupational therapy (Gillis et al., 2003). Meaningful participation and active engagement lead to improved emotional and physical wellbeing in older adults (Eakman et al., 2010; Eakman, 2012), and have been linked to less fatigue and fewer depressive symptoms in healthy adults (Hooker et al., 2020). Thus, this global indicator reflects clinically important behavior. While low motivation would be expected to influence the measure, it presumably also could be affected by other factors, such as mood, cognition, and fatigue. We briefly explored this in our data, finding an influence of cognitive performance, but not anxiety or depression. More work is needed to refine how motivation is assessed in HIV; it might be useful to ground such work in this clinically relevant approach to assessing meaningful goal-directed activity.

Key strengths of this study include a well-characterized large sample, a clinically relevant outcome measure, and pre-registration of the hypotheses and analysis. The study also has limitations. First, only 8 women participated. While this is representative of the current demographics of HIV in Canada and participation in research of women living with HIV in Canada (Mayo et al., 2018; Public Health Agency of Canada, 2020), the results should not be generalized to women until they are replicated in a larger sample. Second, this sample, by design, was made up of people with well-controlled HIV and generally stable overall health. There are reasons to expect differences in brain health in people with poor viral control, which may be associated with ongoing virally mediated brain injury (Sanford et al., 2018a; O’Connor and Zeffiro, 2019). Likewise, current substance use disorder may also affect motivation and feedback-related EEG responses. Such patients were excluded here. Third, the sample size was established for the two BHN sub-studies from which the current work drew, not for this particular study. However, *post hoc* sample size calculations showed that this sample was adequate to detect medium effect sizes in the relationships studied here.

In summary, we found preliminary evidence to support a link between brain responses to feedback measured by FB-P3 and real-world motivation in older people living with chronic HIV infection. Self-reported motivation measured with items from a widely used apathy scale was not linked to any of the EEG correlates of feedback, which may point to problems with this scale, at least for the purposes of studying neural correlates of motivation in HIV. The results here suggest that a promising approach for further neuroscience research on apathy might be to focus on observable motivated behaviors (whether self-reported or clinician-observed) rather than on questionnaires. The FB-P3 has promise as a potential EEG biomarker of motivation, independent of age or nadir CD4 status in HIV. Further work is needed to replicate this result, study the underlying mechanisms, and establish the utility of this EEG marker as a potential biomarker for diagnosis or assessing the effects of interventions.

## Data availability statement

The raw data supporting the conclusions of this article will be made available by the authors, without undue reservation.

## Ethics statement

The studies involving human participants were reviewed and approved by the McGill University Health Centre Research Ethics Board. The patients/participants provided their written informed consent to participate in this study.

## Author contributions

A-LF contributed to the design of the study and collected the data. GC performed the statistical analysis and wrote the first draft of the manuscript. M-JB, NM, and LF contributed to the conception and design of the study, interpretation of the results, and editing of the manuscript. All authors read and approved the submitted version of this manuscript.

## Footnotes

1
https://osf.io/yemhn
