# Supplementary material for: Relationship between reward-related evoked potentials and real-world motivation in older people living with human immunodeficiency virus
Source: Front Aging Neurosci. 2022 Sep 1;14:927209. doi: 10.3389/fnagi.2022.927209 (PMC9475288; doi:10.3389/fnagi.2022.927209)
Supplement: Supplementary file 1 [file Data_Sheet_1.docx]

Supplementary Material

# Supplementary Figures and Tables

**1.1 Supplemental Tables**

**Multiple linear regressions predicting motivation (SAS-R)**

| **Outcome:**  **SAS-R,**  52.04 (13.83) | **Parameter Estimate**  **(*β*)** | **Standard Error** | **95 % CI**  **(lower bound, upper bound)** | **R^2^** | |
| --- | --- | --- | --- | --- | --- |
| **Predictors** |  |  |  |  |  |
| **Gain feedback** | | | | | |
| RewP amplitude, µV | 0.06 | 0.04 | (-0.03, 0.14) |  | |
| Age, decades | -0.21 | 0.15 | (-0.52, 0.10) | 0.06 | |
| FB-P3 amplitude, µV | 0.04 | 0.03 | (-0.02, 0.10) |  | |
| Age, decades | -0.19 | 0.16 | (-0.50, 0.13) | 0.05 | |
| N1 amplitude, µV | -0.01 | 0.05 | (-0.10, 0.09) |  | |
| Age, decades | -0.24 | 0.16 | (-0.55, 0.08) | 0.03 | |
| **Loss feedback** | | | | |  |
| RewP amplitude, µV | 0.06 | 0.04 | (-0.02, 0.15) |  | |
| Age, decades | -0.23 | 0.15 | (-0.53, 0.08) | 0.07 | |
| FB-P3 amplitude, µV | 0.04 | 0.03 | (-0.02, 0.10) |  | |
| Age, decades | -0.20 | 0.16 | (-0.51, 0.12) | 0.06 | |
| N1 amplitude, µV | -0.02 | 0.04 | (-0.10, 0.05) |  | |
| Age, decades | -0.26 | 0.16 | (-0.58, 0.06) | 0.04 | |
| **Difference (loss-gain)** | | | | |  |
| ΔRewP, µV | 0.03 | 0.10 | (-0.17, 0.24) |  | |
| Age, decades | -0.25 | 0.16 | (-0.56, 0.07) | 0.04 | |

**Supplementary Table 1.** Results are from a cluster centered at FCz for RewP and a cluster centered at Pz for FB-P3 gain and loss feedback conditional averages. The ΔRewP was calculated as the difference between loss minus gain feedback, the results shown here are from a cluster centered at FCz. To facilitate interpretation, age is expressed in decades. A logit transformation was applied to SAS-R. ^*^*p* <0.05; ^**^ *p* < 0.01; ^***^ *p* < 0.001

**Multiple linear regressions predicting ERP amplitudes**

| **Outcome,** mean (SD), µV | | **Parameter Estimate**  **(*β*)** | | **Standard Error** | | **95 % CI**  **(lower bound, upper bound)** | | **R^2^** | |
| --- | --- | --- | --- | --- | --- | --- | --- | --- | --- |
| **Predictors** | |  |  |  |  |  |  |  |  |
| **Gain feedback** | | | | | | | | | |
| RewP amplitude, 2.91 (2.39) | |  | | | | | | | |
| Nadir CD4, 100 cells/µL | | -0.21 | | 0.18 | | (-0.57, 0.14) | |  | |
| Age, decades | | -0.54 | | 0.40 | | (-1.33, 0.25) | | 0.04 | |
| FB-P3 amplitude, 6.77 (3.54) | |  | | | | | | | |
| Nadir CD4, 100 cells/µL | | -0.47 | | 0.26 | | (-0.98, 0.05) | |  | |
| Age, decades | | -1.21* | | 0.58 | | (-2.36, -0.06) | | 0.09* | |
| N1 amplitude, -1.70 (2.33) | |  | |  | |  | |  | |
| Nadir CD4, 100 cells/µL | | 0.02 | | 0.17 | | (-0.32, 0.37) | |  | |
| Age, decades | | -0.65 | | 0.39 | | (-1.42, 0.13) | | 0.04 | |
| **Loss feedback** | | | | | | | | | |
| RewP amplitude, 2.18 (2.38) | |  | |  | | | | | |
| Nadir CD4, 100 cells/µL | | -0.09 | | 0.18 | | (-0.45, 0.27) | |  | |
| Age, decades | | -0.22 | | 0.40 | | (-1.02, 0.58) | | 0.01 | |
| FB-P3 amplitude, 6.20 (3.45) | |  | | | | | | | |
| Nadir CD4, 100 cells/µL | | -0.39 | | 0.25 | | (-0.90, 0.12) | |  | |
| Age, decades | | -0.94 | | 0.57 | | (-2.08, 0.20) | | 0.06 | |
| N1 amplitude, -1.06 (3.08) | |  | |  | |  | |  | |
| Nadir CD4, 100 cells/µL | | 0.00 | | 0.20 | | (-0.50, 0.40) | |  | |
| Age, decades | | -1.46 | | 0.49 | | (-2.45, 0.48) | | 0.11 | |
| **Difference (loss-gain)** | | | | | | | | | |
| ΔRewP, -0.73 (1.06) | |  | |  | |  | |  | |
| Nadir CD4, 100 cells/µL | | 0.12 | | 0.08 | | (-0.03, 0.28) | |  | |
| Age, decades | | 0.32 | | 0.17 | | (-0.03, 0.66) | | 0.07 | |

**Supplementary Table 2.** Results are from clusters centered at FCz for RewP and at Pz for FB-P3 . To facilitate interpretation, age is expressed in decades and nadir CD4 count in 100 cells/µL. ^*^ *p* < 0.05; ^**^ *p* < 0.01; ^***^ *p* < 0.0001.
